# Adolescents’ enjoyment in face-to-face physical education during the COVID-19 pandemic

**DOI:** 10.1177/1356336X231163106

**Published:** 2023-03-20

**Authors:** Carlos Mata, Marcos Onofre, João Costa, Diogo Monteiro, Diogo Teixeira, João Martins

**Affiliations:** 70882 Universidade de Lisboa, Portugal; 70882 Universidade de Lisboa, Portugal; 8795 University College Cork, Ireland; 70866 ESECS—Polytechnic of Leiria, Portugal;; Research Center in Sport, Health and Human Development (CIDESD), Portugal;; Life Quality Research Centre (CIEQV), Portugal; 70887 Lusófona University (ULHT/FEFD), Portugal;; Research Center in Sport, Physical Education, and Exercise and Health (CIDEFES), Portugal; 70882 Universidade de Lisboa, Portugal

**Keywords:** Physical education, affective attitude, COVID-19, adolescents, enjoyment

## Abstract

The COVID-19 pandemic has had an impact on the routines of children and adolescents and on their level of involvement in physical activity (PA). The restrictive rules applied in this period affected the functioning of physical education (PE) classes in Portugal, and strongly limited student participation. The aim of this study was to analyze and compare the affective attitude (enjoyment) of adolescents during face-to-face PE lessons during the COVID-19 pandemic (from September 2020), according to sex, education, and PA levels. The study included 1369 students (621 boys and 748 girls), aged 12–18 years, *M*_age_: 14.4; *SD*: 1.74. A validated online questionnaire was distributed between November and December 2020, and the data were analyzed for positive and negative affective attitude, using MANCOVAs (multivariate analysis of covariance) adjusted for sociodemographic and behavioral variables. The results showed higher negative affective attitudes in younger boys when compared to older boys and to girls in the same education level. Younger less active boys also showed higher negative affective attitudes than less active girls.

## Introduction

### Physical education and physical activity during the COVID-19 pandemic

When COVID-19 was declared a global pandemic in March 2020 (WHO, 2020), many countries introduced measures to control the spread of the virus, including social and physical distancing policies, school closures, contact restrictions, and curfews. However, while these measures proved effective in reducing contagions, they led to several negative effects on young people. Some of the negative effects included social isolation, increased sedentary behaviors and screen time, and a significant reduction in opportunities to engage in physical activity (PA) (Chambonniere et al., 2021; Dunton et al., 2020; Nagata et al., 2020; Pombo et al., 2020; Stockwell et al., 2021; Xiang et al., 2020).

Following the lockdown in March 2020, in Portugal, students returned to school in September 2020 to face unprecedented social and physical restrictions that significantly changed their educational experience. For example, restrictions on movement, use of equipment, and social contact became part of daily routines. At the same time, many clubs and sports associations had not reopened, limiting adolescents’ possibilities to engage in PA and sports (Sociedade Portuguesa de Educação Física (SPEF), 2020b). This decrease in PA is worrying, as it worsens the overall trend of unsatisfactory levels of PA in adolescence that was already noticeable before the pandemic (Guthold et al., 2018; Marques et al., 2020; Martins et al., 2019; WHO, 2018). The positive effects of regular engagement in PA for young people are well documented. These include physical, psychological, social, and cognitive benefits (Biddle et al., 2019; Janssen and Leblanc, 2010; Poitras et al., 2016). Hence, this decrease in adolescents’ PA levels during the pandemic should be of special concern.

The school, and especially PE, provides the ideal context for promoting and developing active lifestyles in adolescents (Martins, 2015; Uddin et al., 2020). During the pandemic, PE played a significant role, since it was an opportunity for students to engage in regular (although restricted) PA. However, as schools returned to face-to-face classes following the full lockdown, the functioning of PE lessons was considerably hampered. Health and safety rules were implemented (social distancing; suppression of equipment sharing; reduction of class time due to safety/hygiene protocols), which led to limitations in the use of spaces and class organization (SPEF, 2020b). In most schools in Portugal, team sports were no longer formally performed in PE classes during this period, opting, at best, for reduced or adapted games (SPEF, 2020b). Individual, more fitness-oriented activities became prevalent during face-to-face PE during the pandemic (SPEF, 2020a, 2020b). This restrictive PE environment limited students’ experiences and reduced the chances of effective involvement. Consequently, it did not provide students with the opportunity to access in-depth and enriching learning experiences (Mata et al., 2021; SPEF, 2020b).

### Theoretical basis of attitude: a brief overview

One of the aspects that influences the learning process is attitude (Solmon, 2003) and enhancing positive attitudes impacts learning. Students who feel confident and comfortable in the classroom environment tend to show greater interest in performing a learning task (Mercier et al., 2017; Phillips and Silverman, 2015; Subramaniam and Silverman, 2007). Attitude can be understood as the degree to which behavior is self-assessed (e.g. favorable or unfavorable; positive or negative) and this judgment of the behavior's outcomes will influence the type of behavioral response (Fishbein and Ajzen, 2011; Solmon, 2003; Subramaniam and Mercier, 2017; Subramaniam and Silverman, 2007). For instance, attitude is related to the intention of students to engage in PA participation and may also influence their PA patterns (Subramaniam and Mercier, 2017).

A bi-dimensional view related to attitude comprises affective and cognitive factors (Oppenheim, 1992; Subramaniam and Silverman, 2007), that is, a person's attitude is not only determined by his/her beliefs, since emotions work simultaneously with the cognitive process (Phillips and Silverman, 2015; Subramaniam and Silverman, 2007). The theory of planned behavior (TPB), designed to predict and explain human behavior in different contexts, presents itself as a theoretical model to investigate and understand attitude and how it influences behavior. TPB evolved from the theory of reasoned action (Ajzen, 1980), adding perceived behavioral control (how strongly the individual believes that they can perform a given behavior).

Conceptualization, and, therefore, the measurement of the attitude construct by the characteristics of the outcome perception expectation, results in two distinct types of attitudes: affective attitude—the emotions and perceptions based on affection for a behavior (e.g. boring or pleasant); and instrumental attitude—the cost–benefit perception of a behavior (e.g. worthless or valuable) (Ekkekakis and Brand, 2019; Hagger et al., 2002; Hamilton and Johnson, 2020; Rhodes and Dickau, 2012). The affective/emotional dimension is related to the aspects of fun/pleasure/enjoyment, while cognition refers to the perceived importance or usefulness of the object of attitude (Mercier et al., 2017). Enjoyment can be described as a positive emotional state, which reflects feelings such as pleasure and fun (Scanlan et al., 1993; Wankel, 1993). The positive or negative evaluation that one makes in relation to the behavior determines the intention, that is, the conscious decision to perform the behavior (Ajzen and Madden, 1986; Scanlan et al., 1993; Wankel, 1993).

### Attitude toward PE and PA

Several authors have reported a correlation between pleasure/enjoyment and children's and adolescents’ involvement in PA (Sallis et al., 2000; Van Der Horst et al., 2007). The *fun* dimension emerges as one important criterion to be considered in the pedagogical approach to PE, in order to provide meaningful experiences for students, which translate into successful engagement (Beni et al., 2017).

PE has received special attention, because it can be a lever for children and adolescents to adopt healthy lifestyles (Chen et al., 2014; Sallis et al., 2012; Solmon, 2015). Additionally, PE is an area in which attitude is relevant as it can give valuable indications about the emotions of students (Silverman and Subramaniam, 1999). The development of positive attitudes toward PA may be an influencing factor for young people to remain active outside the school context (Hagger et al., 2003; Solmon, 2003). These factors reinforce the relevance of understanding the extent to which experiences in PE influence attitudes regarding the pursuit of an active lifestyle during childhood and adolescence (Mercier et al., 2017). This perspective is significant given that active adolescents are more likely to adopt active behaviors in adulthood (Subramaniam and Silverman, 2007; Telama et al., 2005).

In this regard, the positive correlation consistently found in studies between attitude toward PA and attitude in PE underlines the importance of providing affirming and enjoyable PA experiences for students. Such experiences are likely to lead to a more favorable attitude and behavioral intention toward PE (Gouveia et al., 2019; Pate et al., 2007; Subramaniam and Silverman, 2007).

In the unique context of the COVID-19 pandemic, it is relevant to understand adolescents’ attitudes in PE lessons, considering all the constraints that were in place. Before the pandemic, there was a tendency for students’ positive attitudes toward PE to decline as they got older, particularly among girls. Previous research shows that, regardless of age group, boys tend to show a more favorable attitude toward PE than girls (Howard et al., 2011; Marques et al., 2014; Phillips and Silverman, 2015; Silverman, 2017; Subramaniam and Silverman, 2007; Zeng et al., 2011). The results of one of the few longitudinal studies in PE, which followed students from a suburban Eastern US school district for 3 years, through 4th–7th and 5th–8th grades, also corroborate this finding, indicating that as grade level increases, attitude decreases, and this is more prevalent in girls than in boys (Mercier et al., 2017). In a mixed-methods study (Subramaniam and Silverman, 2002), the authors emphasize that students who show higher or lower attitudes have distinct experiences in PE, and a repetitive curriculum and perceived usefulness of PE can impact enjoyment and attitude. Affect and curriculum are mentioned by Mercier et al. (2017) as associated with decreasing positive attitudes, supporting the premise that these areas should be targeted by PE teachers, since making lessons fun and enjoyable could have a positive effect on changing students’ attitudes. Furthermore, lack of motor skills and a competition-oriented classroom environment (with team sports prevailing as the primary option in many PE classes) might influence students’ enjoyment and attitude (Bernstein et al., 2011). These issues were accentuated in PE during the COVID-19 pandemic, because of the changes in the way classes operated at this time (e.g. suppression of team games/sports). Thus, considering the context of face-to-face PE during the COVID-19 pandemic and the importance of understanding students’ attitudes during this period, this study aims to analyze adolescents’ attitude, specifically its affective component, with an emphasis on the *enjoyment* dimension, and how it varies by sex, age, and PA level.

## Materials and methods

### Participants and school selection

The study sample consisted of 1369 students (621 boys and 748 girls), aged between 12 and 18 years (*M*_age_: 14.4; *SD*: 1.74), from 13 schools in northern Portugal, belonging to levels two *(lower secondary*) and three (*upper secondary*) of the International Standard of Education (ISCED) classification. The students were from different socioeconomic backgrounds and only those who were attending PE classes were considered eligible for participation in this study.

### Procedures

The *LimeSurvey* platform was used to complete the online questionnaire. The administration took place between November and December 2020, upon the students’ return to face-to-face classes after the lockdown and schools’ closure since March 2020. The online questionnaire was distributed during an adverse context, in the midst of the COVID-19 pandemic, with students experiencing many restrictions that affected the normal functioning of PE lessons: restricted curriculum; reduction of various activities (e.g. team games and gymnastics); limited physical contact and sharing of material and equipment where possible; less class time due to hygiene/safety protocols; mask-wearing by both teachers and students, with most students only removing their masks during more intense activities; adaptation of activities to ensure appropriate 3-m distance between students and classes held in restricted PE spaces; and closed locker rooms (reduced spaces and/or insufficient number) (SPEF, 2020b).

### Data collection

All operational and ethical procedures for the questionnaire administration were met, including contact with the teachers who collaborated in the study (October 2020), to explain the goals and administration process and provision of information to school boards and legal guardians of participating students. Only students duly authorized by guardians (with signed consent) participated in the study and all confidentiality and data protection issues were ensured. Students were informed that they could terminate their participation if they wished. The study was approved by the Ethics Council for Research of the host institution (Opinion No. 16/2020) and by the government department in Portugal that authorizes the administration of surveys in schools (No. 0666900005, approved 03/2020).

Pilot tests were conducted in advance with a group of 15 secondary school students, to assess difficulties in understanding the questions and check how long it took to answer the entire questionnaire. Overall, there were no difficulties in understanding the questions and the average response time to the questionnaire for this group was 25 minutes.

The link to the questionnaire was sent by e-mail to PE teachers and students completed it during PE lessons, in the presence of the PE teacher or another appropriate teacher who was aware of the required procedures. To facilitate the distribution of the questionnaire, the researchers recommended the use of the school's digital devices (computers or tablets).

### Instruments

For the evaluation of enjoyment in PA in young people, Motl et al. (2001) developed the Physical Activity Enjoyment Scale (PACES). Evidence of factorial validity and convergent evidence for construct validity indicates that PACES is a valid instrument to measure enjoyment in PA (Motl et al., 2001). This scale of 16 items has been translated and validated into other languages and/or contexts, including Portugal, where it was validated to assess children's and adolescents’ enjoyment of any PA (Sabino et al., 2019), including the context of PE. This instrument uses a Likert scale, which ranges from 1 (I completely disagree) to 5 (I completely agree) and is composed of nine positive items (e.g., I enjoy it; It's very exciting; It gives me a strong feeling of success) and seven negative ones (e.g. I feel bored; It's no fun at all; It frustrates me), in response to the introductory question “When I participate in PE lessons….” Given the two-factor instrument approach, higher values of positive items coupled with lower values of negative items reflect a higher positive affective attitude, whereas lower values of positive items and higher values of negative items reflect a negative affective attitude (Carraro et al., 2008; Monteiro et al., 2017).

In line with the frequency evaluation of PA by Prochaska et al. (2001), the answers to the two questions regarding the weekly frequency of PA ranged from “0 to 7 days.” The introduction preceding the two questions alluded to the issues students should consider when thinking about PA, depicted as follows:

Physical activity is any activity that increases the heartbeat and makes you pant. Physical activity can be applied to sports, in school activities, playing with friends or walking. Some examples of physical activity are: running, cycling, dancing, skateboarding, swimming, playing basketball, football, and surfing.

Item 2 was adjusted by adding the phrase “before COVID-19,” to determine pre-pandemic PA patterns. **Question 1**: “In the last seven days, how many days have you practiced physical activity for a total of at least 60 minutes per day?”; **Question 2**: “In a normal week, before COVID-19, how many days did you practice at least 60 minutes of physical activity per day?” Similar to previous studies (Mata et al., 2021), students who reported they complied with the WHO recommendation of 60 minutes daily of moderate to vigorous PA (WHO, 2018) were considered “active.”

Five questions were also included to assess age, sex, weight, height, and education level. Body mass index (BMI: kg/m^2^) and BMI *Z*-Score were calculated and adjusted for age and sex, in accordance with the WHO reference values (WHO, 2021).

Socio-economic status (SES) was assessed by the Family Affluence Scale (FAS) (Matos, 2018; Sigmundová et al., 2019; Torsheim et al., 2016). This instrument of six items is a concise asset-based measure of family wealth that was designed to be applied in surveys of adolescents. The six-item limit was based on the assumption that the scale should be feasible for use in large cross-national surveys of school-age children and adolescents, where wide-range coverage would often place limits on the number of items (Torsheim et al., 2016).

The six-item FAS scale replaced the previous four-item FAS scale, incorporating changes in technology and living conditions of recent decades (Hartley et al., 2016; Torsheim et al., 2016). The six-item FAS scale and its answers/scores (with higher scores indicating greater affluence) are as follows: (a) Does your family own a car, van, or truck? (No = 0; Yes, one = 1; Yes, two or more = 2); (b) Do you have your own bedroom to yourself? (No = 0; Yes = 1); (c) How many computers does your family own (including laptops and tablets, not including game consoles and smartphones)? (None = 0; One = 1; Two = 2; More than two = 3); (d) How many bathrooms (room with a bath/shower or both) are there in your home? (None = 0; One = 1; Two = 2; More than two = 3); (e) Does your family have a dishwasher at home? (No = 0; Yes = 1); (f) How many times did you and your family go abroad for a holiday/vacation last year? (Never = 0; Once = 1; Twice = 2; Three or more times = 3).

Question six was replaced with “In the last 12 months, how many times have you made vacation trips with your family?,” due to the travel limitations imposed by the pandemic. A single variable was created from these items, which represents SES.

### Data analysis

The data were automatically organized by the *LimeSurvey* platform into Excel and then exported to SPSS, version 28 to MacOS (SPSS Inc., Chicago, IL), where statistical analysis was performed. In the descriptive analyses, absolute (*n*) and relative (%) frequencies were calculated for categorical variables and means (*M*) and standard deviations (*SD*) for continuous variables. MANCOVAs (multivariate analysis of covariance) were used to compare outcomes, adjusted for age, pre- and post-COVID PA, SES, and BMI (*Z*-score). Three variables were considered for stratification: educational level, sex, and PA (less active/active). When the results were stratified by PA (less active/active), an adjustment was made for the variables of age, pre-COVID PA, SES, and BMI (*Z*-score). In this case, post-COVID PA was not used because it was mutually exclusive to the stratification variable. There were 49 missing cases in the BMI variable (*Z*-score) and therefore in the MANCOVAs the total number of participants was 1320.

To evaluate the assumptions of normality and homoscedasticity of variance, the Kolmogorov–Smirnov and Levene tests were, respectively, performed. In both cases, the assumptions were met. The significance level was set at 0.05.

## Results

### Participant information

The sample description is presented in Table 1, in which we highlight the low percentages of participants who indicated that they complied with the 60 minutes of daily moderate to vigorous PA (WHO, 2018) before the pandemic (4.9%) and during the pandemic (3.1%). Table 2 shows the mean values of the PACES 16-item questionnaire. There is evidence of generally positive attitudes—a higher mean in the positive affective attitude (3.90 ± 0.76), with a lower mean score in negative affective attitude (2.07 ± 0.96).

**Table 1.** Sample description—frequencies (*n*/%) and *M*/*SD*.

	ISCED 2		ISCED 3		TOTAL		AGE		^a^PA		^b^PA	
	*n*	%	*n*	%	*n*	%	*M*	*SD*	*n*	%	*n*	%
Boys	385	62.0	236	38.0	**621**	45.4	14.3	1.74	43	6.9	26	4.1
Girls	432	57.8	316	42.2	**748**	54.6	14.4	1.74	24	3.2	17	2.2
Total	817	59.7	552	40.3	**1369**	100	14.4	1.74	67	4.9	43	3.1

a PA: Performs 60 minutes of PA every day before COVID-19; *M*: mean; *SD*: standard deviation.

b PA: Performs 60 minutes of PA every day during COVID-19; PA: physical activity.

ISCED 2: International Standard of Education, level 2; ISCED 3: International Standard of Education, level 3.

**Table 2.** *M* and *SD* of responses to PACES items.

Item	*When I participate in PE classes…*	Total (*n* = 1369)	
		*M*	*SD*
1	*I enjoy it.*	4.07	0.86
10	*I get something out of it.*	4.05	0.84
11	*It's very exciting.*	3.53	1.01
4	*I find it pleasurable.*	4.01	0.86
6	*It gives me energy.*	3.89	0.97
8	*It's very pleasant.*	3.92	0.90
9	*My body feels good.*	3.91	0.93
14	*It gives me a strong feeling of success.*	3.66	1.01
15	*It feels good.*	4.01	0.86
*Positive Affective Attitude*		**3.90**	**0**.**76**
2	*I feel bored.*	2.14	0.99
3	*I dislike it.*	2.06	1.11
5	*It's no fun at all.*	1.99	1.06
7	*It makes me depressed.*	1.90	1.04
12	*It frustrates me.*	2.02	1.06
13	*It's not at all interesting.*	1.99	1.07
16	*I feel as though I’d rather be doing something else.*	2.38	1.15
*Negative Affective Attitude*		**2.07**	**0**.**96**

PACES—Physical Activity Enjoyment Scale; *M*—mean; *SD*—standard deviation.

### Comparison between sex, stratified by level of education

An association between negative affective attitude and sex is observed at ISCED 2 (*p* = 0.001). The results suggest that negative affective attitude scores are higher in boys (2.21 ± 0.05) when compared to girls (1.98 ± 0.05) in ISCED 2, although the recorded levels of this type of attitude are considered relatively low, that is, they reflect a positive attitude in general. At this level of education, there were no differences in the positive affective attitude dimension. At the ISCED 3 level, there were no significant statistical differences in the level of positive and negative affective attitude between sex (Table 3).

**Table 3.** MANCOVA: comparison by sex, stratified by level of education.

	ISCED 2			ISCED 3		
*Outcomes*	Boys	Girls	*p*-Value	Boys	Girls	*p*-Value
	(*n* = 380)	(*n* = 429)		(*n* = 214)	(*n* = 297)	
Positive affective attitude	3.87 (0.04)	3.94 (0.04)	*p* = 0.219	3.94 (0.05)	3.87 (0.04)	*p* = 0.243
Negative affective attitude	2.21 (0.05)	1.98 (0.05)	***p* = 0.001**	2.01 (0.07)	2.03 (0.06)	*p* = 0.775

Results adjusted for age, pre- and post-COVID-19 PA, SES, and BMI (*Z*-score).

ISCED 2: International Standard of Education, level 2; ISCED 3: International Standard of Education, level 3.

Results with statistical significance are presented in bold.

### Comparison by education cycle, stratified by sex

Higher levels of negative affective attitude were found in ISCED 2 compared to ISCED 3, but only in males (*p* < 0.001). Younger boys showed a higher tendency toward negative affective attitudes (2.34 ± 0.07) than older boys (1.70 ± 0.12). Among boys, there were no significant differences in positive affective attitude. In the comparative analysis between girls, at both levels of education, there were no significant differences in positive and negative affective attitude levels (Table 4).

**Table 4.** MANCOVA: comparison by school year, stratified by sex.

	Boys			Girls		
*Outcomes*	ISCED 2	ISCED 3	*p*-Value	ISCED 2	ISCED 3	*p*-Value
	(*n* = 380)	(*n* = 214)		(*n* = 429)	(*n* = 297)	
Positive affective attitude	3.86 (0.05)	4.08 (0.08)	*p* = 0.053	3.92 (0.05)	3.80 (0.07)	*p* = 0.244
Negative affective attitude	2.34 (0.07)	1.70 (0.12)	***p* < **0**.001**	2.04 (0.06)	2.00 (0.08)	*p* = 0.728

Results adjusted for age, pre- and post-COVID-19 PA, SES, and BMI (*Z*-score).

Results with statistical significance are presented in bold.

### Comparison between sex, stratified by level of physical activity

In the less active group, the negative affective attitude was higher in boys (*p* = 0.043), when compared to girls. In active students, there were no differences between boys and girls (Table 5).

**Table 5.** MANCOVA: comparison by sex, stratified by less active and active.

	Less active			Active		
*Outcomes*	Boys	Girls	*p*-Value	Boys	Girls	*p*-Value
	(*n* = 570)	(*n* = 709)		(*n* = 24)	(*n* = 17)	
Positive affective attitude	3.90 (0.03)	3.88 (0.03)	*p* = 0.685	4.41 (0.15)	4.17 (0.18)	*p* = 0.347
Negative affective attitude	2.13 (0.04)	2.02 (0.04)	***p* = 0.043**	1.99 (0.27)	1.89 (0.32)	*p* = 0.825

Results adjusted for age, pre- and post-COVID-19 PA, SES, and BMI (*Z*-score).

Results with statistical significance are presented in bold.

## Discussion

This study aimed to investigate the affective attitudes of students in PE during the COVID-19 pandemic, when Portuguese schools returned to face-to-face classes with significant curricular, pedagogical, and organizational constraints imposed by health and safety regulations. While the overall attitude can be presented as positive, boys showed a tendency toward higher negative affective attitudes, particularly the younger and less active ones.

In terms of PA involvement, and this includes PE, before the pandemic, 4.9% of the participants reported being physically active, that is, they complied with the WHO (2018) PA guidelines in adolescence. This percentage is in line with the values reported in the Portuguese national study by Baptista et al. (2012), who used objective evaluation measures (accelerometers), and below the results presented by Martins et al. (2019), HBSC (2014), and WHO (2018), which used self-reporting questionnaires. The percentage of adolescents who reported that they met the recommendations dropped to 3.1% during the pandemic. In both situations, girls reported lower percentage values of PA involvement than boys, in line with the findings of other studies, which report lower PA levels in girls (Baptista et al., 2012; Guthold et al., 2018; Marques et al., 2020; Martins et al., 2019).

Several studies report the tendency for negative health indicators during the pandemic in children and adolescents, namely, those related to a decrease in PA involvement and increased time in sedentary activities (Pombo et al., 2020; Stockwell et al., 2021; Xiang et al., 2020). These studies highlight schools, and especially PE, as favorable contexts to increase the level of PA and to contribute to the promotion of healthy lifestyles (Martins et al., 2022) and physical literacy (Martins et al., 2021) in the young population. During the COVID-19 pandemic, PE in Portugal, in most cases, became the only setting where adolescents had the opportunity to engage in PA (SPEF, 2020b).

In this study, younger boys (ISCED 2) showed significantly higher levels of negative affective attitude in PE lessons than girls. Furthermore, when compared to older boys (ISCED 3), younger boys revealed higher levels of negative affective attitude. There is a tendency for students’ positive attitudes in PE to decrease as they get older and this tends to happen faster in girls than in boys (Silverman, 2017). The results suggest a greater negative impact of the pandemic on younger boys, which may be explained by the difficulties they experienced in adapting to the restrictive context of PE classes and the decrease in enjoyment they may have felt as a result of potentially less appealing lessons: for example, mandatory distance between students and the reduction or suppression of team games. The more individualized and fitness-oriented activities that became more prevalent in face-to-face PE classes during the pandemic may have also negatively affected the less active boys, as they showed higher levels of negative affective attitude when compared to girls in the same group. No significant differences were found in this study between the groups under analysis on the positive affective attitude dimension. However, when comparing boys to girls in ISCED 2, girls showed a higher value in positive affective attitude. Several studies suggest that boys have a more positive attitude toward PE than girls (Hünük and Demirhan, 2010; Koca et al., 2005; Zeng et al., 2011). However, in this study, the differences were not noticeable. The pandemic did not affect everyone in the same way, which may explain why this difference was not found in this study. It may be the case that girls in this study did not like PE more, but rather the boys were more impacted by the less appealing classes during the COVID-19 pandemic. This is an important consideration, since the negative evaluation (less enjoyment) of a behavior may imply less involvement in the class and a less favorable attitude (Mercier et al., 2017). The possible lack of meaningful experiences—fun, social interaction, challenge, motor competence, and relevant personal learning (Beni et al., 2017)—in PE lessons during the pandemic is a factor of concern, due to the future negative implications that may result from it. We should take note of the data found in this and other studies that address PA issues during the pandemic, as future events of this type may occur, and it will be important to provide favorable conditions for adolescents to stay active and motivated in PE classes.

It will be important to investigate what attitudes students reveal in PE classes after the pandemic and to what extent the experiences during the pandemic have impacted on the way students engage and have fun in PE classes. Studying attitude in PE, and the factors that influence it, is essential if we want adolescents to value PE and become physically active individuals throughout their lives (Silverman, 2017). Therefore, in terms of future research, we recommend an examination of students’ attitudes toward PE after the pandemic, focused on understanding the extent to which students’ affective attitudes toward PE return to pre-pandemic standards, and if the change in PE lessons will be sufficient for students (especially for younger boys) to express the enjoyment they showed before COVID-19. It will also be necessary for PE teachers to be aware of the impacts of the pandemic on students’ attitudes so that their pedagogical interventions can reverse this negative effect, and their classes provide an enjoyable environment full of meaningful experiences (O’Brien et al., 2020).

This study primarily sought to analyze differences between groups, with a convenience sample, which is a limitation. The study has a cross-sectional design, so it does not allow for a cause-and-effect relationship between the COVID-19 pandemic and the students’ attitudes to be obtained. PA was assessed by self-report, which may lead to less accurate results, when compared to objective measurements, especially in young people.

The sample size and originality of this study are its strengths. It should be noted that most studies conducted during the pandemic were either in the context of online PE classes, focused on PE teachers’ experiences during lockdown or intended to describe the impact on children's and adolescents' PA levels during the pandemic (Gobbi et al., 2020; Howley, 2022). This study, on the other hand, refers to a very particular period, after the return to school, in a timeframe in which the number of infections began to rise, preceding the second lockdown in Portugal, which would happen in January 2021.

The study of attitudes in PE classes is of the utmost importance. After a period when students had to adapt to a difficult context, it will be important to investigate what attitudes students reveal after their return to normalcy. Considering that another pandemic may occur, it raises the importance of alerting policy-makers to the need to adopt measures to promote PA in the school context that generate positive attitudes among students. This study was conducted during an unusual period, so the results cannot be compared to a “normal” context. Still, we underline from this study the association between the COVID-19 pandemic and the less positive affective attitude in face-to-face PE classes.

## Conclusions

In the context of face-to-face PE during the pandemic, the changes in students’ routines seem to have had a negative association with enjoyment of PE. This seems to have been more visible in boys, especially in younger and less active ones. Before the pandemic, younger and more active boys showed positive attitudes toward PA and PE. They appear to have been the group of students most affected by the restrictions that were imposed on PE lessons during this critical period. Considering these factors, the promotion of an active lifestyle gains even greater significance at the time of returning to normalcy. Given the increase in sedentary behaviors and reduction in the involvement of children and adolescents in PA during the pandemic, school and PE may play an essential role in counteracting this trend.

## References

[bibr1-1356336X231163106] Ajzen I Madden TJ (1986) Prediction of goal-directed behavior: Attitudes, intentions, and perceived behavioral control. Journal of Experimental Social Psychology 22(5): 453–474.

[bibr2-1356336X231163106] Ajzen IFM (1980) Understanding Attitudes and Predicting Social Behavior. Englewood Cliffs, NJ: Prentice-Hall.

[bibr3-1356336X231163106] Baptista F Santos DA Silva AM, et al. (2012) Prevalence of the Portuguese population attaining sufficient physical activity. Medicine & Science in Sports & Exercise 44(3): 466–473. 21844823 10.1249/MSS.0b013e318230e441

[bibr4-1356336X231163106] Beni S Fletcher T Ní Chróinín D (2017) Meaningful experiences in physical education and youth sport: A review of the literature. Quest (Grand Rapids, Mich ) 69(3): 291–312.

[bibr5-1356336X231163106] Bernstein E Phillips SR Silverman S (2011) Attitudes and perceptions of middle school students toward competitive activities in physical education. Journal of Teaching in Physical Education 30(1): 69–83.

[bibr6-1356336X231163106] Biddle SJH Ciaccioni S Thomas G, et al. (2019) Physical activity and mental health in children and adolescents: An updated review of reviews and an analysis of causality. Psychology of Sport and Exercise 42: 146–155.

[bibr7-1356336X231163106] Carraro A MC Y Robazza C (2008) A contribution to the validation of the physical activity enjoyment scale in an Italian sample. Social Behavior and Personality: An International Journal 36(7): 911–918.

[bibr8-1356336X231163106] Chambonniere C Lambert C Fearnbach N, et al. (2021) Effect of the COVID-19 lockdown on physical activity and sedentary behaviors in French children and adolescents: New results from the ONAPS national survey. European Journal of Integrated Medicine 43: 101308. 10.1016/j.eujim.2021.101308 PMC7871771 33584872

[bibr9-1356336X231163106] Chen S Sun H Zhu X, et al. (2014) Relationship between motivation and learning in physical education and after-school physical activity. Research Quarterly for Exercise and Sport 85(4): 468–477. 25412129 10.1080/02701367.2014.961054 PMC9161286

[bibr10-1356336X231163106] Dunton G Do B Wang S (2020) Early effects of the COVID-19 pandemic on physical activity and sedentary behavior in children living in the U.S. BMC Public Health 20: 1351. 32887592 10.1186/s12889-020-09429-3 PMC7472405

[bibr11-1356336X231163106] Ekkekakis P Brand R (2019) Affective responses to and automatic affective valuations of physical activity: Fifty years of progress on the seminal question in exercise psychology. Psychology of Sport and Exercise 42: 130–137.

[bibr12-1356336X231163106] Fishbein M Ajzen I (2011) Predicting and Changing Behavior: The Reasoned Action Approach. New York, NY: Psychology Press.

[bibr13-1356336X231163106] Gobbi E Maltagliati S Sarrazin P, et al. (2020) Promoting physical activity during school closures imposed by the first wave of the COVID-19 pandemic: Physical education teachers’ behaviors in France, Italy and Turkey. International Journal of Environmental Research and Public Health 17(24): 9431. 33339228 10.3390/ijerph17249431 PMC7767079

[bibr14-1356336X231163106] Gouveia ÉR Ihle A Gouveia BR, et al. (2019) Students’ attitude toward physical education: Relations with physical activity, physical fitness, and self-concept. The Physical Educator 76(4): 945–963.

[bibr15-1356336X231163106] Guthold R Stevens GA Riley LM, et al. (2018) Worldwide trends in insufficient physical activity from 2001 to 2016: A pooled analysis of 358 population-based surveys with 1·9 million participants. Lancet Global Health 6(10): e1077–e1086. 30193830 10.1016/S2214-109X(18)30357-7

[bibr16-1356336X231163106] Hagger M Chatzisarantis N Biddle S (2002) A meta-analytic review of the theories of reasoned action and planned behavior in physical activity: Predictive validity and the contribution of additional variables. Journal of Sport & Exercise Psychology 24: 3–32.

[bibr17-1356336X231163106] Hagger MS Chatzisarantis NLD Culverhouse T, et al. (2003) The processes by which perceived autonomy support in physical education promotes leisure-time physical activity intentions and behavior: A trans-contextual model. Journal of Educational Psychology 95: 784–795.

[bibr18-1356336X231163106] Hamilton K Johnson BT (2020) 31 attitudes and persuasive communication interventions. The Handbook of Behavior Change 445–460.

[bibr19-1356336X231163106] Hartley JEK Levin K Currie C (2016) A new version of the HBSC family affluence scale – FAS III: Scottish qualitative findings from the international FAS development study. Child Indicators Research 9(1): 233–245. 26925177 10.1007/s12187-015-9325-3 PMC4757604

[bibr20-1356336X231163106] HBSC (2014) Health of Portuguese adolescents during recession: data from 2014 national report. Available at: http://aventurasocial.com/arquivo/1437158618_RELATORIO%20HBSC%202014e.pdf.

[bibr21-1356336X231163106] Howard ZZ Raymond MH L W (2011) Attitudes of High School Students toward Physical Education and Their Sport Activity Preferences. Journal of Social Sciences 7(4): 529–537.

[bibr22-1356336X231163106] Howley D (2022) Experiences of teaching and learning in K-12 physical education during COVID-19: An international comparative case study. Physical Education and Sport Pedagogy 27(6): 608–625.

[bibr23-1356336X231163106] Hünük D Demirhan G (2010) Turkish adolescents’ attitudes toward physical education. Perceptual and Motor Skills 111(2): 324–332. 21162436 10.2466/06.07.11.13.PMS.111.5.324-332

[bibr24-1356336X231163106] Janssen I Leblanc AG (2010) Systematic review of the health benefits of physical activity and fitness in school-aged children and youth. International Journal of Behavioral Nutrition and Physical Activity 7: 40. 20459784 10.1186/1479-5868-7-40 PMC2885312

[bibr25-1356336X231163106] Koca C Aşçı FH Demirhan G (2005) Attitudes toward physical education and class preferences of Turkish adolescents in terms of school gender composition. Adolescence 40(158): 365–373. 16114598

[bibr26-1356336X231163106] Marques A Henriques-Neto D Peralta M, et al. (2020) Prevalence of physical activity among adolescents from 105 low, middle, and high-income countries. International Journal of Environmental Research and Public Health 17(9): 3145. 32365969 10.3390/ijerph17093145 PMC7246732

[bibr27-1356336X231163106] Marques A Martins J Santos F, et al. (2014) Correlates of school sport participation: A cross-sectional study in urban Portuguese students. Science & Sports 29(4): e31–e38.

[bibr28-1356336X231163106] Martins J (2015) Physical education and lifestyles: Why are teens physically (in)active? Doctoral Thesis. Faculty of Human Kinetics, University of Lisbon, Lisbon.

[bibr29-1356336X231163106] Martins J Marques A Gouveia ÉR, et al. (2022) Participation in physical education classes and health-related behaviours among adolescents from 67 countries. International Journal of Environmental Research and Public Health 19(2): 955. 35055777 10.3390/ijerph19020955 PMC8775417

[bibr30-1356336X231163106] Martins J Marques A Loureiro N, et al. (2019) Trends and age-related changes of physical activity among Portuguese adolescent girls from 2002–2014: Highlights from the health behavior in school-aged children study. Journal of Physical Activity and Health 16(4): 281–287. 30862244 10.1123/jpah.2018-0092

[bibr31-1356336X231163106] Martins J Onofre M Mota J, et al. (2021) International approaches to the definition, philosophical tenets, and core elements of physical literacy: A scoping review. Prospects 50(1): 13–30.

[bibr32-1356336X231163106] Mata C Onofre M Costa J, et al. (2021) Motivation and perceived motivational climate by adolescents in face-to-face physical education during the COVID-19 pandemic. Sustainability 13(23): 13051.

[bibr33-1356336X231163106] Matos MSAT (2018) The health of Portuguese adolescents after the recession. Health Behaviour in School Aged Children (HBSC) study report in (ebook).

[bibr34-1356336X231163106] Mercier K Donovan C Gibbone A, et al. (2017) Three-Year study of Students’ attitudes toward physical education: Grades 4–8. Research Quarterly for Exercise and Sport 88(3): 307–315. 28661718 10.1080/02701367.2017.1339862

[bibr35-1356336X231163106] Monteiro D Nunes G Marinho DA, et al. (2017) Translation and adaptation of the physical activity enjoyment scale (PACES) in a sample of Portuguese athletes, invariance across genders nature sports and swimming. Revista Brasileira de Cineantropometria & Desempenho Humano 19: 631–643.

[bibr36-1356336X231163106] Motl RW Dishman RK Saunders R, et al. (2001) Measuring enjoyment of physical activity in adolescent girls. American Journal of Preventive Medicine 21(2): 110–117. 11457630 10.1016/s0749-3797(01)00326-9

[bibr37-1356336X231163106] Nagata JM Abdel Magid HS Pettee Gabriel K (2020) Screen time for children and adolescents during the coronavirus disease 2019 pandemic. Obesity 28(9): 1582–1583. 32463530 10.1002/oby.22917 PMC7283714

[bibr38-1356336X231163106] O’Brien W Adamakis M O’Brien N, et al. (2020) Implications for European physical education teacher education during the COVID-19 pandemic: A cross-institutional SWOT analysis. European Journal of Teacher Education 43(4): 503–522.

[bibr39-1356336X231163106] Oppenheim A (1992) Questionnaire Design, Interviewing, and Attitude Measurement. London: Bloomsbury Publishing.

[bibr40-1356336X231163106] Pate RR Ward DS O’Neill RJ, et al. (2007) Enrollment in physical education is associated with overall physical activity in adolescent girls. Research Quarterly for Exercise and Sport 78(4): 265–270. 17941531 10.1080/02701367.2007.10599424

[bibr41-1356336X231163106] Phillips SR Silverman S (2015) Upper elementary school student attitudes toward physical education. Journal of Teaching in Physical Education 34(3): 461–473.

[bibr42-1356336X231163106] Poitras VJ Gray CE Borghese MM, et al. (2016) Systematic review of the relationships between objectively measured physical activity and health indicators in school-aged children and youth. Applied Physiology, Nutrition, and Metabolism 41(6): S197–S239. 10.1139/apnm-2015-0663 27306431

[bibr43-1356336X231163106] Pombo A Luz C Rodrigues LP, et al. (2020) Correlates of children's physical activity during the COVID-19 confinement in Portugal. Public Health 189: 14–19. 33126117 10.1016/j.puhe.2020.09.009 PMC7508519

[bibr44-1356336X231163106] Prochaska JJ Sallis JF Long B (2001) A physical activity screening measure for use with adolescents in primary care. Archives of Pediatrics & Adolescent Medicine 155(5): 554–559. 11343497 10.1001/archpedi.155.5.554

[bibr45-1356336X231163106] Rhodes RE Dickau L (2012) Experimental evidence for the intention–behavior relationship in the physical activity domain: A meta-analysis. Health Psychology 31(6): 724–727. 22390739 10.1037/a0027290

[bibr46-1356336X231163106] Sabino B Almeida MJ Fonseca A (2019) Adaptação, Validação e Avaliação da Invariância de escalas de medida intrapessoal relacionadas com a atividade física para o contexto escolar português. Retos: Nuevas Tendencias en Educación Física, Deporte y Recreación 36: 87–91.

[bibr47-1356336X231163106] Sallis JF McKenzie TL Beets MW, et al. (2012) Physical education's role in public health: Steps forward and backward over 20 years and HOPE for the future. Research Quarterly for Exercise and Sport 83(2): 125–135. 22808697 10.1080/02701367.2012.10599842 PMC6036633

[bibr48-1356336X231163106] Sallis JF Prochaska JJ Taylor WC (2000) A review of correlates of physical activity of children and adolescents. Medicine & Science in Sports & Exercise 32(5): 963–975. 10795788 10.1097/00005768-200005000-00014

[bibr49-1356336X231163106] Scanlan TK Carpenter PJ Lobel M, et al. (1993) Sources of enjoyment for youth sport athletes. Pediatric Exercise Science 5(3): 275–285.

[bibr50-1356336X231163106] Sigmundová D Sigmund E Tesler R, et al. (2019) Vigorous physical activity in relation to family affluence: Time trends in Europe and North America. International Journal of Public Health 64(7): 1049–1058. 31278436 10.1007/s00038-019-01271-8 PMC6677706

[bibr51-1356336X231163106] Silverman S (2017) Attitude research in physical education: A review. Journal of Teaching in Physical Education 36(3): 303–312.

[bibr52-1356336X231163106] Silverman S Subramaniam PR (1999) Student attitude toward physical education and physical activity: A review of measurement issues and outcomes. Journal of Teaching in Physical Education 19(1): 97–125.

[bibr53-1356336X231163106] Solmon MA (2003) Student issues in physical education classes: Attitudes, cognition, and motivation. Student Learning in Physical Education: Applying Research to Enhance Instruction 2: 147–164.

[bibr54-1356336X231163106] Solmon MA (2015) Optimizing the role of physical education in promoting physical activity: A social-ecological approach. Research Quarterly for Exercise and Sport 86(4): 329–337. 26558638 10.1080/02701367.2015.1091712

[bibr55-1356336X231163106] SPEF C (2020a) Proposals for face-to-face delivery of practical sessions of PE and School Sports – School Year 2020–2021.

[bibr56-1356336X231163106] SPEF C (2020b) Survey on the functioning of Physical Education and School Sports. What are the conditions for Physical Education and School Sports in your School in times of pandemic COVID-19?

[bibr57-1356336X231163106] Stockwell S Trott M Tully M, et al. (2021) Changes in physical activity and sedentary behaviours from before to during the COVID-19 pandemic lockdown: A systematic review. BMJ Open Sport & Exercise Medicine 7(1): e000960. 10.1136/bmjsem-2020-000960 PMC7852071 34192010

[bibr58-1356336X231163106] Subramaniam P Mercier K (2017) Attitudes matter in physical education. International Journal of Physical Education 54(4): 22–30.

[bibr59-1356336X231163106] Subramaniam P Silverman S (2002) Using complimentary data: An investigation of student attitudes in physical education. Journal of Sport Pedagogy 8: 74–91.

[bibr60-1356336X231163106] Subramaniam PR Silverman S (2007) Middle school students’ attitudes toward physical education. Teaching and Teacher Education 23(5): 602–611.

[bibr61-1356336X231163106] Telama R Yang X Viikari J, et al. (2005) Physical activity from childhood to adulthood: A 21-year tracking study. American Journal of Preventive Medicine 28(3): 267–273. 15766614 10.1016/j.amepre.2004.12.003

[bibr62-1356336X231163106] Torsheim T Cavallo F Levin KA, et al. (2016) Psychometric validation of the revised family affluence scale: A latent Variable approach. Child Indicators Research 9: 771–784. 27489572 10.1007/s12187-015-9339-x PMC4958120

[bibr63-1356336X231163106] Uddin R Salmon J Islam S, et al. (2020) Physical education class participation is associated with physical activity among adolescents in 65 countries. Scientific Reports 10: 22128. 33335213 10.1038/s41598-020-79100-9 PMC7746694

[bibr64-1356336X231163106] Van Der Horst K Paw MJ Twisk JW, et al. (2007) A brief review on correlates of physical activity and sedentariness in youth. Medicine & Science in Sports & Exercise 39(8): 1241–1250. 17762356 10.1249/mss.0b013e318059bf35

[bibr65-1356336X231163106] Wankel LM (1993) The importance of enjoyment to adherence and psychological benefits from physical activity. International Journal of Sport Psychology 24(2): 151–169.

[bibr66-1356336X231163106] WHO (2018) Global action plan on physical activity 2018–2030: more active people for a healthier world. Available at: https://apps.who.int/iris/handle/10665/272722.

[bibr67-1356336X231163106] WHO (2020) WHO interactive timeline: WHO’s COVID-19 response. Available at: https://www.who.int/emergencies/diseases/novel-coronavirus-2019/interactive-timeline.

[bibr68-1356336X231163106] WHO (2021) Growth reference data for 5–19 years Indicators/BMI-for-age (5–19 years).

[bibr69-1356336X231163106] Xiang M Zhang Z Kuwahara K (2020) Impact of COVID-19 pandemic on children and adolescents’ lifestyle behavior larger than expected. Progress in Cardiovascular Diseases 63(4): 531–532. 32360513 10.1016/j.pcad.2020.04.013 PMC7190470

[bibr70-1356336X231163106] Zeng HZ Hipscher M Leung RW (2011) Attitudes of high school students toward physical education and their sport activity preferences. Journal of Social Sciences 7(4): 529.

